# Distinct recruitment of human eIF4E isoforms to processing bodies and stress granules

**DOI:** 10.1186/s12867-016-0072-x

**Published:** 2016-08-30

**Authors:** Klara Frydryskova, Tomas Masek, Katerina Borcin, Silvia Mrvova, Veronica Venturi, Martin Pospisek

**Affiliations:** 1 Laboratory of RNA Biochemistry, Department of Genetics and Microbiology, Faculty of Science, Charles University in Prague, Viničná 5, 128 00 Prague 2, Czech Republic; 2 Centre for Genomic Regulation (CRG), The Barcelona Institute of Science and Technology, Dr. Aiguader 88, 08003 Barcelona, Spain

**Keywords:** Eukaryotic translation initiation factor 4E (eIF4E), eIF4E2, eIF4E3, Processing body (P-body), Stress granule, Translation initiation factor, Translation control, Heat shock, Arsenite, PB, SG

## Abstract

**Background:**

Eukaryotic translation initiation factor 4E (eIF4E) plays a pivotal role in the control of cap-dependent translation initiation, modulates the fate of specific mRNAs, occurs in processing bodies (PBs) and is required for formation of stress granules (SGs). In this study, we focused on the subcellular localization of a representative compendium of eIF4E protein isoforms, particularly on the less studied members of the human eIF4E protein family, eIF4E2 and eIF4E3.

**Results:**

We showed that unlike eIF4E1, its less studied isoform eIF4E3_A, encoded by human chromosome 3, localized to stress granules but not PBs upon both heat shock and arsenite stress. Furthermore, we found that eIF4E3_A interacts with human translation initiation factors eIF4G1, eIF4G3 and PABP1 in vivo and sediments into the same fractions as canonical eIF4E1 during polysome analysis in sucrose gradients. Contrary to this finding, the truncated human eIF4E3 isoform, eIF4E3_B, showed no localization to SGs and no binding to eIF4G. We also highlighted that eIF4E2 may exhibit distinct functions under different stresses as it readily localizes to P-bodies during arsenite and heat stresses, whereas it is redirected to stress granules only upon heat shock. We extended our study to a number of protein variants, arising from alternative mRNA splicing, of each of the three eIF4E isoforms. Our results surprisingly uncovered differences in the ability of eIF4E1_1 and eIF4E1_3 to form stress granules in response to cellular stresses.

**Conclusion:**

Our comparison of all three human eIF4E isoforms and their protein variants enriches the intriguing spectrum of roles attributed to the eukaryotic initiation translation factors of the 4E family, which exhibit a distinctive localization within different RNA granules under different stresses. The localization of eIF4E3_A to stress granules, but not to processing bodies, along with its binding to eIF4G and PABP1 suggests a role of human eIF4E3_A in translation initiation rather than its involvement in a translational repression and mRNA decay and turnover. The localization of eIF4E2 to stress granules under heat shock but not arsenite stress indicates its distinct function in cellular response to these stresses and points to the variable protein content of SGs as a consequence of different stress insults.

**Electronic supplementary material:**

The online version of this article (doi:10.1186/s12867-016-0072-x) contains supplementary material, which is available to authorized users.

## Background

In eukaryotes, the mRNA 5′-cap structure (m^7^GpppN) is recognized by translation initiation factor 4E (eIF4E, hereinafter referred to as eIF4E1), which brings the mRNAs to the ribosome via an interaction with scaffold protein eIF4G [1]. Cap recognition occurs through two conserved tryptophan residues (W56 and W102 in human eIF4E1) that sandwich the 7-methylguanosine moiety [2]. eIF4E1 is regulated by both the phosphorylation of Ser209 and interactions with eIF4G, eIF4E-binding proteins (4E-BPs) and the eIF4E transporter (4E-T), which bind eIF4E through a phylogenetically conserved region (S/T)V(E/D)(E/D)FW on its convex side [3–6]. The availability of eIF4E—and ultimately the formation of a functional mammalian eIF4F complex—is affected by the translational repressors 4E-BPs. Discovered as a molecular mimic of eIF4G, the 4E-BP family of proteins competes for the same eIF4E1 binding motif, thereby inhibiting the initiation of protein synthesis [7]. The substitution of W73 to a non-aromatic residue in human eIF4E1 leads to its inability to interact with 4E-BPs or eIF4G [6] and to be recruited to sites of mRNA degradation and stress-induced RNA cytoplasmic granules [8]. Conversely, substitution of the cap-binding W56 and W102 (W100 and W146 in *Drosophila* eIF4E-1) to non-aromatic residues in canonical eIF4E1 does not affect its localization to these cytoplasmic foci. Therefore, interactions of eIF4E1 with its protein partners rather than its cap-binding ability seem to be essential for eIF4E1 relocalization to the stress-induced RNA cytoplasmic granules [8]. Although eIF4E1 is predominantly localized to the cytoplasm, a substantial fraction of eIF4E1 can move to the nucleus via the importin α/β pathway by virtue of its interaction with the nucleocytoplasmic shuttling protein 4E-T [5, 9]. Tight control of eIF4E1 activity and localization is crucial for cellular growth and survival, as witnessed by its contribution to malignancy. Overexpression of eIF4E1 leads to an oncogenic transformation, and increased eIF4E1 levels are observed in diverse tumor types [10].

In eukaryotes, the complexity of the translation initiation machinery and associated regulatory networks substantially increased during evolution. Metazoans evolved several paralogous eIF4E genes that encode distinctly featured proteins, which are, in addition to regular translation initiation, involved in the preferential translation of particular mRNAs or are tissue and/or developmental stage specific. The number of paralogous genes coding for eIF4E proteins, mostly belonging to the class 1 family, is unprecedentedly high in some organisms e.g., eight such genes have been found in *Drosophila* and five in *Caenorhabditis* [11–13] (for reviews see e.g. [13–16]). In this report we will focus exclusively on human eIF4E protein isoforms and their variants. In addition to eIF4E1 (class 1 member encoded by human chromosomes 4, 5 and 17), members of class 2 (eIF4E2) and class 3 (eIF4E3) of the eIF4E protein family are encoded by the human genome [17]. Both eIF4E2 (encoded by human chromosome 2) and eIF4E3 (encoded by human chromosome 3) are capable of cap-binding, albeit with a 40-fold lower affinity in comparison to eIF4E1, and they further differ in their abilities to bind eIF4G and 4E-BPs [18–20]. eIF4E2 is involved in the translational repression of specific mRNAs, rather than in global translation [21–24]. Under defined circumstances such as hypoxia, however, it can participate in active translation in human cells [25, 26]. The nematode eIF4E2 isoform (IFE-4) was also shown to participate in translation initiation of a small subset of worm’s mRNAs. A substantial part of these mRNAs encode proteins directly or indirectly involved in egg laying [27]. eIF4E2 is also known to be part of a gene expression signature underlying an ability of solid primary tumors to form metastases [28]. Limited data is available with regard to eIF4E3, which contains a cysteine in a position equivalent to aromatic W56 of human eIF4E1 [19] and thus binds the cap via an atypical mode, replacing the aromatic sandwich by multiple Van der Waals interactions [20]. eIF4E3 mRNA level is low in hypoxic breast cancer cells, whereas the eIF4E1 level is up-regulated by hypoxia-inducible factor 1α [10]. Thus, eIF4E3 might underlie an important inhibitory mechanism that is lost in high-level eIF4E1 cancers [20]. Another recent study, however, ascribed an active role in translation initiation to mouse eIF4E3 [29].

eIF4E1 is a component forms a part of RNA granules called processing bodies (PBs) and represents a functionally essential component of stress granules (SGs) [9, 30–34]. SGs emerge as a direct consequence of the stress-induced phosphorylation of eIF2α or by inhibition of assembly and/or function of the eIF4F complex [35–39]. They are extremely dynamical structures found to be juxtaposed or to interact with PBs [40, 41]. SGs contain components of the active translation machinery (e.g., eIF4F, eIF3, small ribosomal subunits, poly(A+)-mRNA) and, in this regard, differ from PBs, where many proteins of the mRNA repression and degradation machinery, components of the RNA interference pathway, proteins involved in nonsense-mediated decay and non-polyadenylated mRNA are warehoused [41–43]. Targeting of mRNA from polysomes to PBs or SGs presumes mRNP remodeling [40, 44–46]. The small repressor polypeptides 4E-BPs tightly bind eIF4E1 in the nucleus, although upon heat stress, its unbound pool shifts from the nucleus to the cytoplasm, where it localizes to SGs. Curiously, this phenomenon has not been observed in arsenite-treated cells [47, 48].

The present study focuses on the less characterized members of the human eIF4E protein family. We cloned cDNA coding for several human variants of all three eIF4E classes and determined their localization to PBs and SGs. Full-length eIF4E3 (eIF4E3_A) never co-localizes with PBs but recruits to SGs in both heat-shocked and arsenite-treated cells, which is in sharp contrast to the observations made on prototypical eIF4E1. eIF4E2 localized to PBs both under arsenite and heat stress and ascertained in SGs upon high temperature but not arsenite treatment. We also detected a significant interaction of eIF4E3_A with components of the translation initiation complex, eIF4G and PABP, and its loading to the monosome and light polysome fractions. This observation strongly suggests a possible active role of eIF4E3_A in translation. Truncated variant eIF4E3_B localizes neither to SG nor to PBs and it does not interact with eIF4G and PABP, which clearly suggests its distinct cellular role from eIF4E3_A. In addition to our findings shedding some light on the new roles of translation initiation factors from the eIF4E family, this work generally emphasizes the importance of investigating protein variants that appear as a result of alternative transcription initiation and post-transcriptional events.

## Methods

### RNA isolation, RT-PCR and cloning

cDNA coding for all the transcription isoforms of eIF4E proteins described in this study were obtained from the REH pre-B leukemic cell line, except the eIF4E3_A variant. RNA was purified according to a modified Chomczynski and Sacchi method [49] from 7–10 × 10^6^ cells. DNase I treatment and reverse transcription were performed with the RNase-Free DNase Set (Qiagen) and the iScript™ cDNA Synthesis Kit (Bio-Rad), respectively. PCR was carried out by the FastStart High Fidelity PCR System (Roche). Amplified cDNA was inserted into the pCR2.1 vector using the TOPO TA Cloning Kit (Invitrogen).

A two-step procedure was employed to clone eIF4E3_A. First, a partial coding sequence covering amino acids 1–215 was amplified from the HEK293-cell-derived cDNA and inserted into the pCR4-TOPO vector (Invitrogen). Next, a missing part of the coding sequence was obtained from the pEGFP-C1-eIF4E3_B vector, prepared as described below. These two segments were joined through the Eco130I restriction site.

To determine the subcellular localization of the eIF4E isoforms, the pEGFP-C1 vector (CMV-IE promoter, Clontech) was utilized to enable the ectopic expression of the particular eIF4E variants as N-terminal GFP fusions. Recombinant vectors were created by cloning eIF4E fragments cut by either BglII/SalI or SalI/Acc65I restriction endonucleases into pEGFP-C1 plasmid. All newly created plasmids were verified by sequencing. A list of transcript variants coding for the eIF4E proteins and primers used for their cloning is available in Table 1.

**Table 1:** The eIF4E isoforms used throughout this study and primers used to clone the corresponding cDNA

Gene	Protein isoform	Primer sequence		Restriction site	Protein variant (Acc. No./GI)	Encoded by transcript variant (Acc. No./GI):	
eIF4E1	1	fwd	CGAAGA**AGATCT**ATGGCGACTGTCGAACCGG	BglII	NP_001959.1 GI:4503535	1	NM_001968.3 GI:194578905
		rev	GCGC**GGTACC**TTAAACAACAAACCTATTTTTAGTGGTGG	Acc65I			
	3	fwd	TTGTCGACATGTTGGACCTGACCTCCCGC	SalI	NP_001124150.1 GI:194578907	3	NM_001130678.1 GI:194578906
		rev	GCGC**GGTACC**TTAAACAACAAACCTATTTTTAGTGGTGG	Acc65I			
eIF4E2	A	fwd	GT**GTCGAC**ATGAACAACAAGTTCGACGCTTTGAAAGATG	SalI	NP_004837.1 GI:4757702	1	NM_004846.3 GI:545309374
		rev	GGGTACCTCATGGCACATTCAACCGCGGCTTC	Acc65I			
	C	fwd	GT**GTCGAC**ATGAACAACAAGTTCGACGCTTTGAAAGATG	SalI	NP_001263265.1 GI:545309640	3	NM_001276336.1 GI:545309639
		rev	CC**GGTACC**TCACAATGTGATTTTTGTATTTCGAAAGC	Acc65I			
	CRA_a	fwd	GT**GTCGAC**ATGAGTTTGAAAGATGATGAC	SalI	EAW71007.1 GI:119591413		not annotated yet
		rev	CC**GGTACC**TCACAATGTGATTTTTGTATTTCGAAAGC	Acc65I			
eIF4E3	A*	fwd	CGGAGAAAATGGCGCTGCCC	X	NP_001128123.1 GI:197382708	1	NM_001134651.1 GI:197382707
		rev	ACTGTGGACGTGCTGTCCTTGG	X			
	B	fwd	GT**GTCGAC**ATGAGAGGAGAGAGGCGACCACTTTG	SalI	NP_775495.1 GI:27659734	2	NM_173359.4 GI:197382627 NM_001134649.2 GI:544583504 NM_001134650.1 GI:197382663 NM_001282886.1 GI:544583491
					NP_001128121.1 GI:197382649	3	
					NP_001128122.1 GI:197382664	4	
		rev	CC**GGTACC**TTAGTGTTTTCCACGTCCACCTTCAAAAG	Acc65I	NP_001269815.1 GI:544583492	5	

* Indicates primers used for the amplification of the 3′-incomplete coding sequence of eIF4E3_A from HEK293-cell cDNA; the complete ORF was obtained in subsequent cloning steps

### Cell culture

Human B cell precursor leukemia cell line REH (t12;21, carries *ETV6*-*RUNX1* fusion) was maintained in RPMI-1640 medium (Gibco) supplemented with 10 % FBS, 2 mM l-glutamine and 1 % antibiotic/antimycotic solution (Gibco) at 5 % CO_2_. Human osteosarcoma U2OS and human embryonic kidney 293 (HEK293) cell lines were maintained in DMEM (Gibco) supplemented with 10 % FBS, 2 mM l-glutamine at 5 % CO_2_.

### Transfection

Transient transfections of U2OS cells were performed using Turbofect transfection reagent (Fermentas) in Opti-MEM^®^ I Reduced-Serum Medium (Gibco). For the immunofluorescence experiments, cells were plated in six-well plates 4 h before transfection. Transfection reactions were carried out by mixing 4 μg of DNA with 6 μl of Turbofect in a total volume of 400 μl of Opti-MEM. The average transfection efficiency was 40 %.

### Oxidative stress

A stock solution of sodium arsenite (Sigma-Aldrich, 35000-1L-R) was diluted to 1 mM concentration in medium preheated to 37 °C shortly before use; cells were treated for 40 min.

### Heat stress

Pre-warmed medium was added to cell cultures and the cultivation dish was immediately placed on a preheated thermoblock. Temperature was measured directly on the coverslip, inside the dish, using a submersible probe and a digital thermometer (Testo 735-2; Additional file 1: Figure S1).

**Fig. 1:**
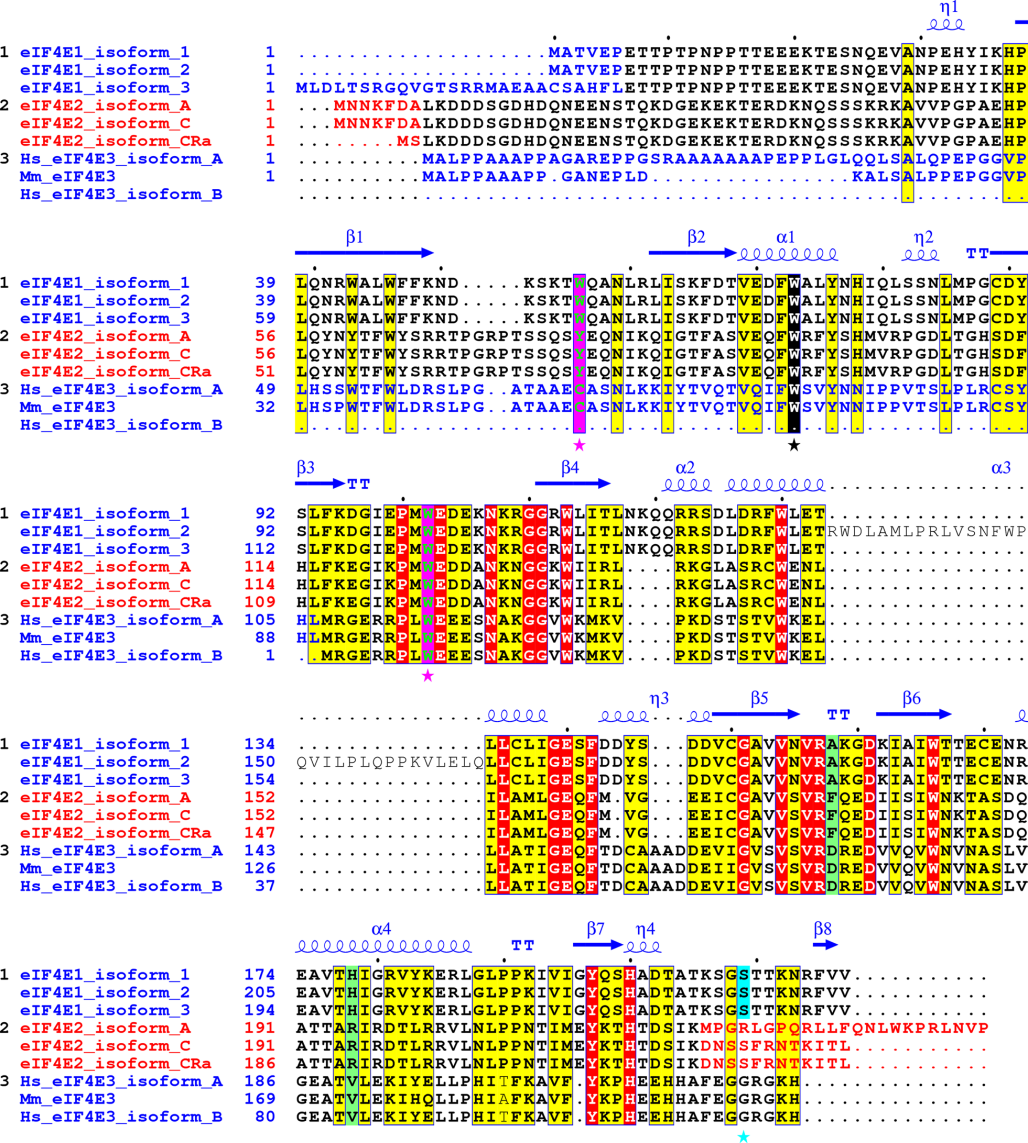
Alignment of the human eIF4E1, eIF4E2 and eIF4E3 isoforms and their variants explored in this study. *Yellow* and *red boxes* denote amino acids high similarity and identity, respectively. Utilization of alternative exons coding for different N- and C-protein termini is marked with *blue* and *red letters*. Cap-binding residues W56 and W102 in eIF4E1 and corresponding amino acids in eIF4E2 and eIF4E3 are highlighted as *green letters* in *purple boxes* and marked with *purple asterisks*. The conserved W73 (on the basis of eIF4E1_1) is marked with *black box* and *black asterisk*. Ser^209^ in eIF4E1 (numbering as of eIF4E1_1) is shaded in *turquoise blue*. Mouse eIF4E3 was added to highlight differences in primary structure between mouse and human orthologs. PDB file 3AM7 was used to depict eIF4E1 secondary structure [56]

### Antibodies

The following antibodies were used throughout the whole study: mouse monoclonal to eIF3η C-5 (Santa Cruz Biotechnology, sc-137114, 1:500), rabbit polyclonal to DDX6 (Bethyl Laboratories, A300-461A, 1:500), rabbit polyclonal to eIF4E1 (Sigma-Aldrich, E5906, 1:200), rabbit polyclonal to eIF4E2 (Genetex, GTX82524, 1:200), rabbit polyclonal to 4E-T (kind gift from Prof. Sonenberg, 1:200), Cy™3-conjugated anti-mouse antibody and Cy™5-conjugated anti-rabbit antibody (Jackson ImmunoResearch Laboratories, 715-165-151, 711-175-152, 1:500), mouse monoclonal anti-β-actin antibody (A2228, Sigma-Aldrich, 1:1000), mouse monoclonal anti-GFP antibody (sc-9996, Santa Cruz, 1:1000), mouse monoclonal anti-eIF4G1 antibody (sc-373892, Santa Cruz, 1:500) and goat anti-mouse IgG-HRP (sc-2005, Santa Cruz, 1:5000).

### Immunofluorescence and microscopy

Cells were seeded on glass coverslips, washed with PBS and fixed in 4 % paraformaldehyde for 18 min at 19–24 h post-transfection. Samples were permeabilized, blocked, sequentially probed with primary and secondary antibodies and finally mounted in ProLong Gold Antifade mounting medium (Invitrogen). Images were captured using an inverted confocal microscope Leica TCS SP2 with an Acousto-Optical Beam Splitter (AOBS) and/or Cell-R system on an inverted Olympus IX81 microscope and UPLSAPO 60× objective. Images were then compiled using ImageJ (Fiji 1,48b) and a graphics editor.

### GFP-Trap immunoprecipitation, mass spectrometry and western blot analyses

Cell lysates of transiently transfected HEK293 cells from 90 % confluent 100 mm dish were harvested 40 h after transfection. Cell lysis and immunoprecipitation with GFP–Trap_A beads were performed as instructed by the manufacturer (ChromoTek). Input (corresponding to 2–3 × 10^5^ cells), bound (corresponding to 1.2–1.5 × 10^6^ cells in one lane) and unbound protein fractions were separated by 12 % SDS-PAGE and visualized with either Coomassie blue stain or Western blot. For MS analysis, bands of interest were excised and processed according to the method described in [50]. MS spectra were acquired on a 4800 Plus MALDI TOF/TOF analyzer (AB Sciex). Mascot server 2.2.07 and a current release of the SWISS-PROT human database were employed for peptide identification with the following settings: carbamidomethylation as fixed; methionine oxidation and N, Q deamination as variable modifications; one missed cleaving site allowed; precursor accuracy at 50 ppm; MS spectrum accuracy at 0.25 Da. Cell lines Flp-In™ T-REx™ 293 (Life Technologies) stably producing respective proteins were used for western blot analyses. Western blotting was performed as described previously [51], with the exceptions of using a PVDF membrane for protein transfer (Biorad) and the ImageQuant LAS4000 Series imaging system (GE Healthcare Life Sciences) for chemiluminescence signal acquiring.

### m^7^GTP-agarose pull-down

Two 100 mm fully confluent dishes of HEK293 cells were transfected with pEGFP-C1-eIF4E1_1 vector using polyethylenimine. Twenty-four hours post transfection, cells were lysed in 300 μl of the GFP-Trap^®^ lysis buffer, enriched with 1 mM PMSF and Complete, EDTA-free^®^ protease inhibitor coctail, centrifuged and sonicated. Eight µl of the lysate was directly loaded to the gel as input. The remaining part of the lysate was incubated with 40 µl of m^7^GTP agarose resin from Jenna Bioscience (AC-142L) at 4 °C on the rotating wheel for 1 h. The resin was washed with 3 × 1 ml of GFP-Trap^®^ washing buffer and subsequently boiled with 24 µl of 2× PAGE loading buffer; 8 µl of the sample was loaded on the gel.

### Polysome profile analysis

Hek293 cell lines stably expressing EGFP or corresponding EGFP-fusion proteins were grown to 60–70 % confluency in 150 mm diameter dishes. Cells were washed by ice-cold PBS and lysed in 10 mM Hepes, pH 7,5; 62,5 mM KCl; 5 mM MgCl_2_; 2 mM DTT; 1 % Triton X-100; 100 μg/ml cycloheximide; Complete EDTA-free (Roche, 1 tablet/10 ml); and 40 U/ml Ribolock (Fermentas). Lysates were cleared by centrifugation in 10,000×*g* for 5 min at 4 °C and then cast on 10–50 % sucrose gradients, which were prepared in solution containing 10 mM Hepes, pH 7,5; 100 mM KCl; 5 mM MgCl_2_; 2 mM DTT; 100 μg/ml cycloheximide; Complete EDTA-free (1 tablet/100 ml); and 5 U/ml Ribolock (Fermentas). Ultracentrifugation and polysome profile analysis were done according [52]. Profiles were fractionated to 10 equal fractions; proteins were purified by TCA—isopropanol procedure and dissolved in 1× Laemmli buffer supplemented with 50 mM TCEP (Sigma-Aldrich) and 1× Complete protease inhibitor cocktail.

## Results

### Cloning of cDNAs encoding two distinct eIF4E1, three eIF4E2 and two eIF4E3 isoforms from REH and HEK293 human cell lines

**Fig. 2:**
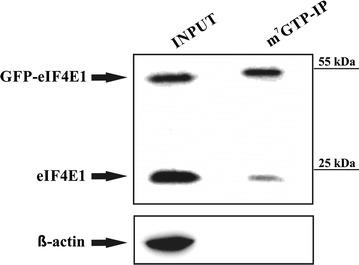
GFP-eIF4E1 fusion protein is capable to bind to m^7^GTP agarose. HEK293 cells were lysed (INPUT) and the lysate was incubated with the m^7^GTP-agarose. Western blot was developed with anti-eIF4E1 antibody, which clearly shows that both endogenous eIF4E1 and its GFP-eIF4E1 fusion counterpart retain their ability to bind the m^7^G cap. Actin was used as a control of a sufficient washing of the m^7^GTP affinity resin

An evolutionary conserved protein core of eIF4E1 is required to mediate its cap-binding activity, interactions with protein partners and its localization into RNA granules. Transcript variants encoding eIF4E family members currently deposited in GenBank and RefSeq databases primarily differ in their 5′-proximal and 3′-terminal coding exons (Fig. 1). The effect of variable eIF4E1 N- and C-termini on its cellular localization and protein–protein interactions remains, however, to be addressed. The concurrent presence of several splicing variants and corresponding protein isoforms within one tissue also remains elusive. We cloned coding regions of the main eIF4E isoforms using cDNA from the leukemic cell line REH, which is derived from B cell precursors bearing *ETV6*-*RUNX1* fusion. We successfully cloned the coding sequences of two prototypical members of the eIF4E family: eIF4E1_1 (protein isoform 1, GI: 4503535), which has extensively been used for structural studies [53–55], and eIF4E2_A (protein isoform A, GI: 4757702). In addition to the canonical eIF4E1 and eIF4E2 proteins, we cloned the coding sequences of the following isoforms: eIF4E1_3 (protein isoform 3, GI: 194578907), which possesses a longer N-terminus; eIF4E2_C (protein isoform C, GI: 545309640), which utilizes a different 3′-distal exon harboring a protein with a shorter C-terminus; and eIF4E2_CRA_a (protein isoform CRA_a), which is not covered by any RefSeq record, even though it is available under GI: 119591413 in the GenBank. This protein isoform has a different N-terminus, though it retains a C-terminus identical to eIF4E2_C. In the case of eIF4E3, five transcript variants are reported in the RefSeq database, although they encode only two protein isoforms. Transcript variants 2 to 5 encode isoform B, which lacks the N-terminal region of the eIF4E3_A prototype. Despite numerous RT-PCR experimental attempts, we were unable to detect a full-length eIF4E3_A transcript in the REH cell line, but we succeeded in its cloning from HEK293 cells. Altogether, we did not prove the existence of mRNAs coding for protein isoforms eIF4E1_2 (GI: 194578909), eIF4E2_D (GI: 545309210) or eIF4E2_X3 (GI: 530371207), despite the use of appropriate primer sets. A detailed description of eIF4E variants used throughout this work is depicted in Fig. 1 and Table 1. To be able to obtain more insight into the function of different eIF4E isoforms and their variants and to overcome lack of suitable antibodies on the market, we prepared a larger set of mammalian expression vectors allowing for the production of eIF4E proteins tagged with GFP on their N-termini. This approach allowed us to study some so far unstudied eIF4E isoforms and to see certain dynamics of their relocalization in cells upon insults such as heat shock and arsenite treatment. The ability of fusion proteins to recognize their natural cellular partners was confirmed by immunoprecipitation followed by western blotting against known binders and/or by mass spectrometry. In the case of eIF4E1, which specifically recognizes the mRNA cap during translation initiation, full functionality of the GFP fusion protein ectopically produced in human cells was confirmed by its efficient binding to the affinity resin containing an immobilized m^7^GTP (Fig. 2). As evidenced further in this study, GFP-eIF4E1 and GFP-eIF4E3_A sedimented with monosome and light polysome fractions similarly as the endogenous eIF4E1 protein. Such confirmations of fusion protein functionality can be extrapolated to all the eIF4E isoforms because all of them contain an unstructured N-terminus and share a high level of sequence and structural similarity of the protein core (Fig. 1).

### Following heat shock, eIF4E2 is found in both PBs and SGs, whereas eIF4E3_A relocates only to SGs

In light of recent publications showing that eIF4E1 is a part of both PBs and SGs [31–34, 39, 48], we were interested whether eIF4E2 or eIF4E3 are also components of RNA granules. To provoke SGs formation, we followed published reports and incubated living cells seeded in the dish for 30 min in a water bath preheated at 44 °C [48]. Possibly due to a range of physical and biological factors such as different cell lines and thicknesses of the dish and/or coverslips, we did not succeed in SGs induction. We therefore decided to incubate the cells on a preheated thermoblock and to measure the temperature in the medium using a digital thermometer with a submersible probe directly touching the coverslip. This novel approach reproducibly led to SGs induction and permitted accurate experiment to experiment comparisons. SGs induction was effective within a narrow temperature range (39.5–42.7 °C), upon which a middle value, 41.7 °C, was opted for further experimentation. Higher temperatures did not lead to immediate cellular death; however, the assembly of SGs was no longer observed (Additional file 1: Figure S1).

Throughout the whole study, SGs were detected as cytoplasmic foci accumulating eIF3B using immunostaining and confocal microscopy. The translation initiation factor eIF3B is a well-accepted SG marker which is specific for stress granules and doesn’t localize to PBs [41]. As a PB marker, we chose DDX6 (rck/p54) DEAD-box helicase. DDX6 is an essential constituent of PBs and its siRNA-induced knock-down leads to the disassembly of PBs and release of their content into the cytoplasm [57]. Wilczynska et al. reported that under certain circumstances DDX6 localized both to PBs and SGs in HeLa cells [58]. On the basis of this report, some authors classify DDX6 as a PB/SG marker. Later on, Souquere et al. analysed the presence of DDX6 in PBs and SGs in HeLa cells subjected to various stresses using confocal microscopy and immunoelectron microscopy. They concluded that DDX6 localization provides an unambiguous detection of PBs [59]. More recently, a comprehensive proteomic study on PBs assembly revealed DDX6 among three essential proteins which were required for PBs assembly in all the conditions tested [43]. Throughout the whole study we never observed DDX6 localizing to SGs, regardless of the stress conditions applied.

**Fig. 3:**
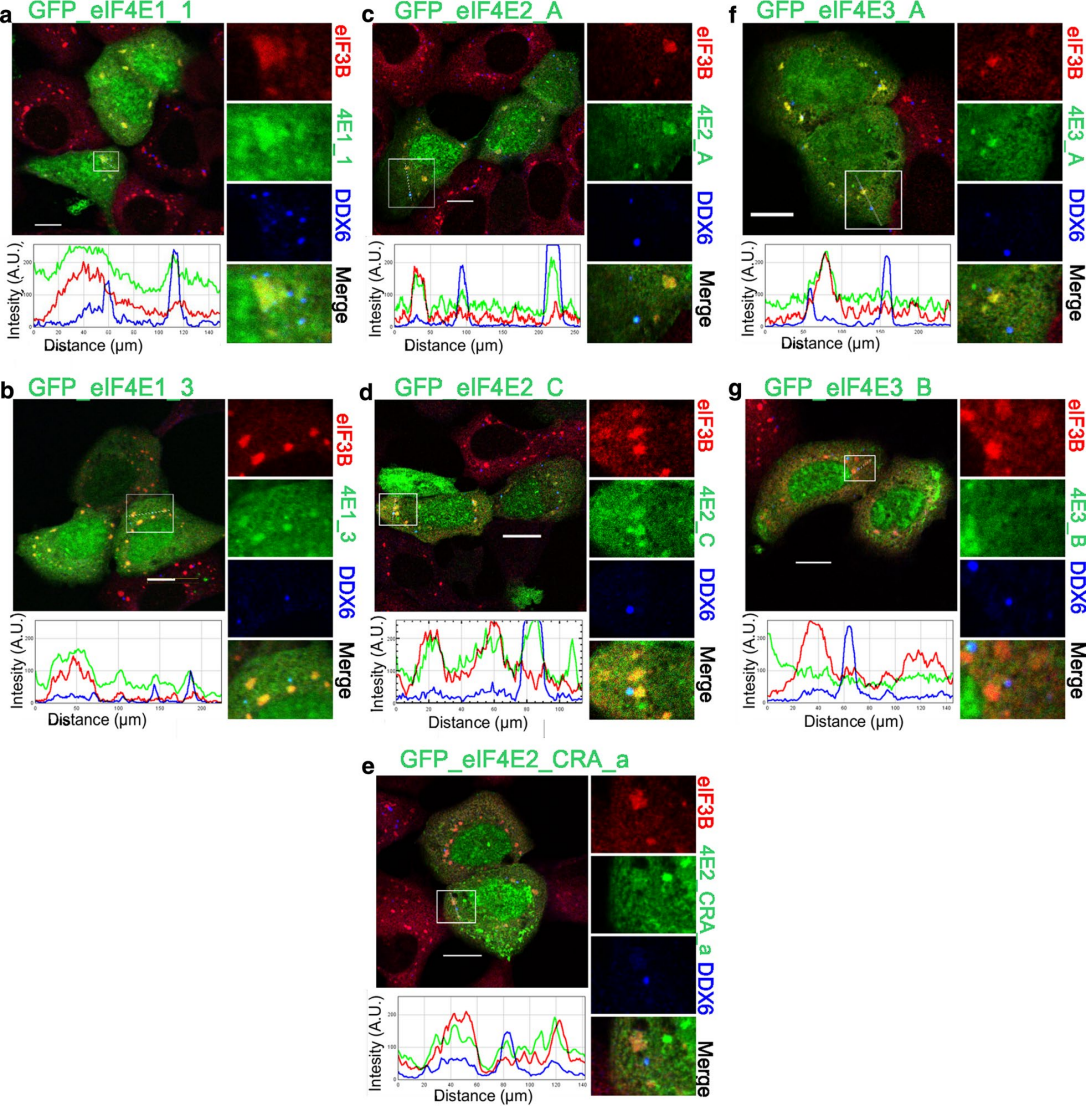
Co-localization of the eIF4E isoforms with PBs and SGs during heat shock. The eIF4E1, 2, 3 proteins (*green*) were ectopically produced in fusion with GFP in U2OS cells. Nineteen hours after transfection, the cells were exposed to 41.7 °C for 30 min, fixed and assessed for eIF3B-stained SGs (*red*) and DDX6-stained PBs (*blue*). Co-localization of the particular eIF4E with SGs and PBs is demonstrated in the* boxed area* replicated in higher magnification on the *right side* of each panel and by the intensity profile measured along the *dashed white line* within the *boxed area*. Both eIF4E1 (**a**, **b**) and all three eIF4E2 (**c**– **e**) variants co-localized with SGs and PBs. The eIF4E3_A (**f**) was recruited only to SGs, and eIF4E3_B (**g**) co-localized with neither SGs nor PBs. Approximately 50 cells transfected with either vector were observed in two independent biological replicates. *Scale bar*, 20 µm

**Fig. 4:**
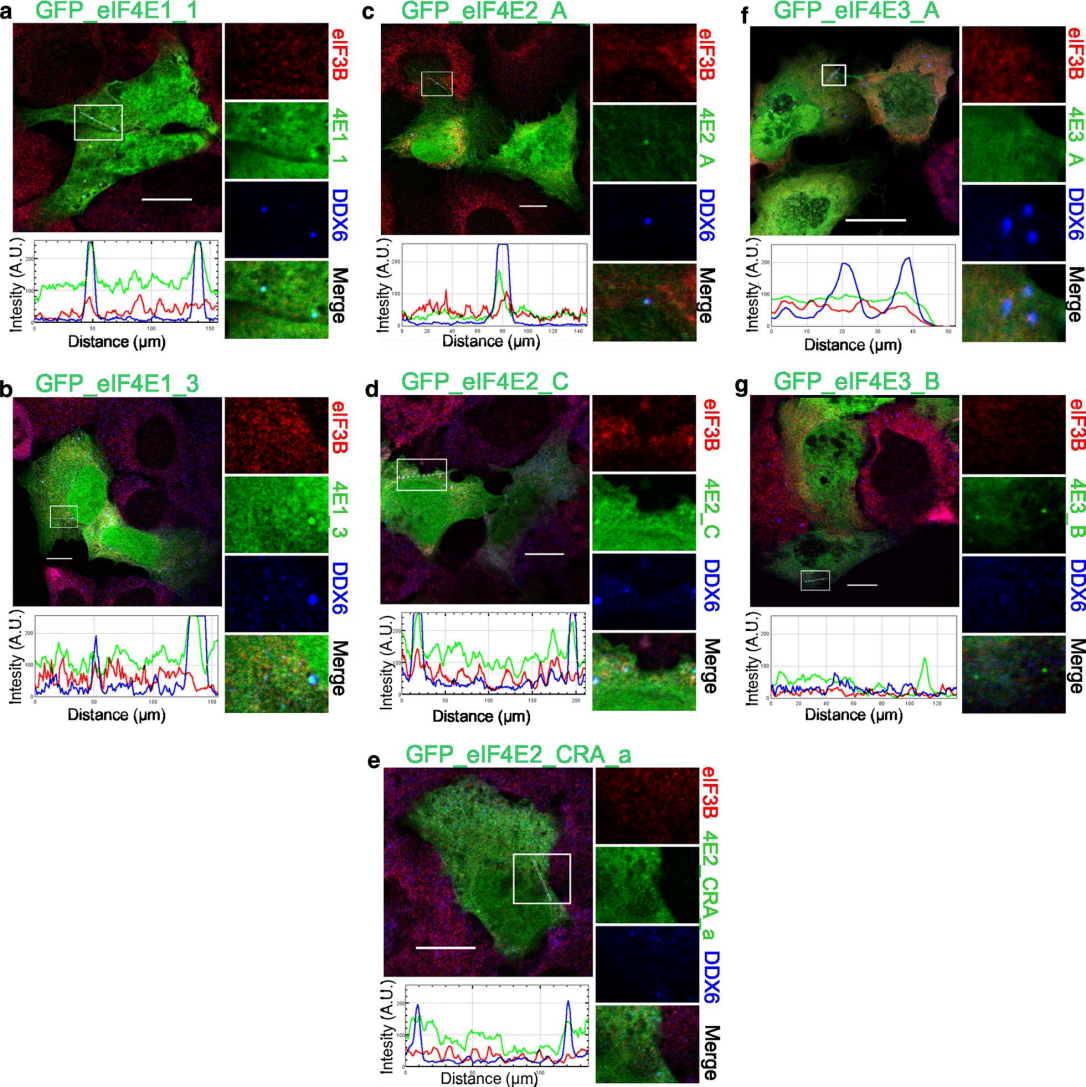
Co-localization of the eIF4E isoforms with PBs in control stress-free cells. The eIF4E1, 2, 3 proteins (*green*) were ectopically produced in fusion with GFP in U2OS cells. Nineteen hours after transfection, the cells were fixed and assessed for eIF3B-stained SGs (*red*) and DDX6-stained PBs (*blue*). No development of stress granules was observed. Co-localization of the particular eIF4E with PBs is demonstrated in the *boxed area* replicated in higher magnification on the* right side* of each panel and by the intensity profile measured along the *dashed white line* within the *boxed area*. Both eIF4E1 (**a**, **b**) and all three eIF4E2 (**c**–**e**) variants co-localized with PBs. No co-localization with PBs was detected for eIF4E3_A (**f**) or eIF4E3_B (**g**). Approximately 50 cells transfected with each plasmid were investigated in two independent biological replicates. *Scale bar*, 20 µm

Following a 30-min heat exposure, all ectopically expressed GFP-eIF4E2 variants co-localized with PBs and, intriguingly, also with SGs (Fig. 3c, d, e), similarly to both GFP-eIF4E1 variants (Fig. 3a, b). GFP-eIF4E3_A was found only within SGs and was never observed in PBs (Fig. 3f); GFP-eIF4E3_B isoform was not detected in either SGs or PBs (Fig. 3g). No stress granules formed inside the stress-free cells, and only the co-localization of all GFP-eIF4E1 and GFP-eIF4E2 proteins with P-bodies could be detected. No co-localization of GFP-eIF4E3 with PBs was observed in untreated control cells (Fig. 4).

### Different localization of eIF4E2 and eIF4E3_A upon arsenite-induced oxidative stress

**Fig. 5:**
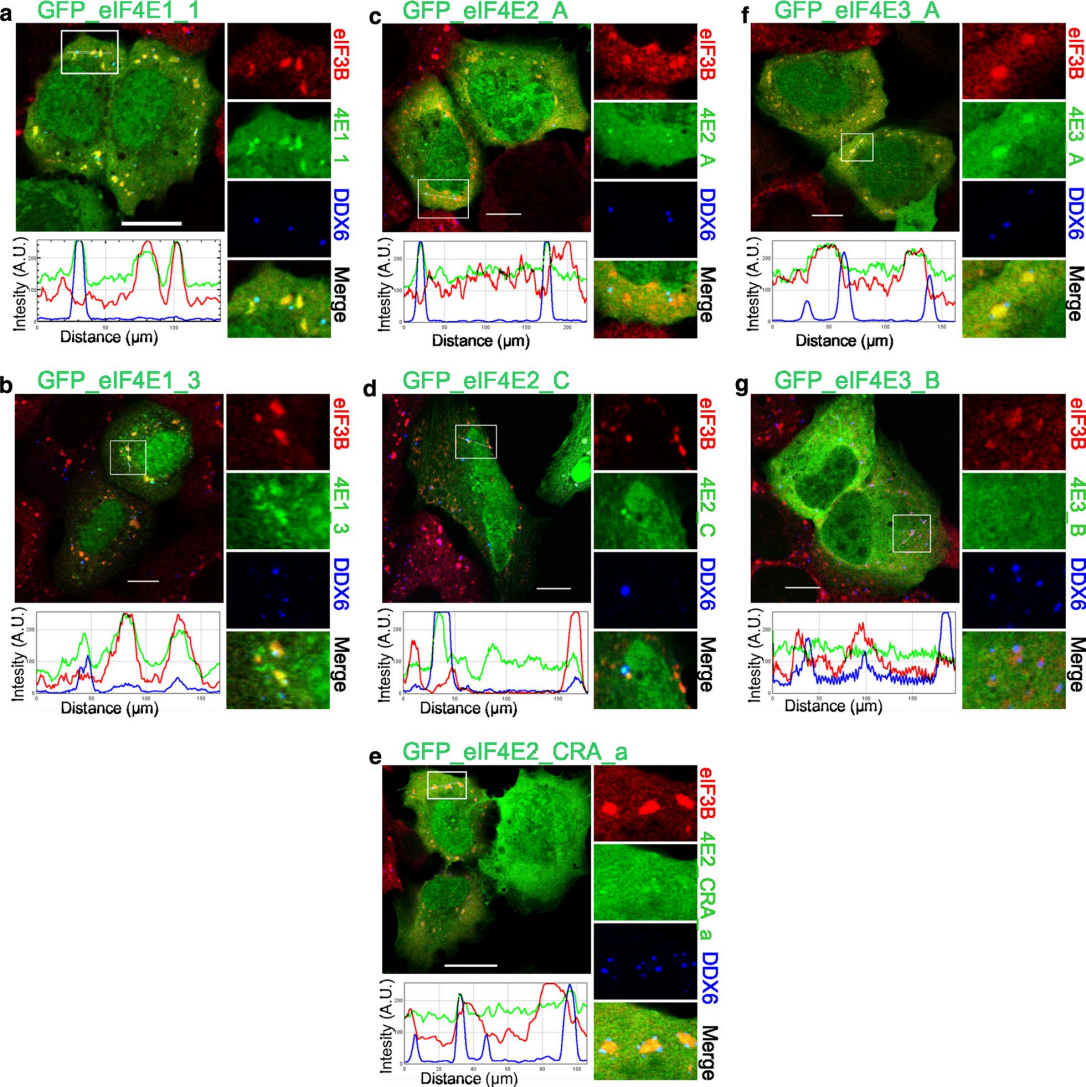
Co-localization of the eIF4E proteins and their isoforms with PBs and SGs during oxidative stress. The eIF4E1, 2, 3 proteins (*green*) were ectopically produced in fusion with GFP in U2OS cells. Nineteen hours post-transfection, the cells were treated with 1 mM sodium arsenite for 40 min, fixed and assessed for eIF3B-stained SGs (*red*) and DDX6-stained PBs (*blue*). Co-localization of the particular eIF4E with SGs and PBs is demonstrated in the *boxed area* on the *right side of each panel* and by the intensity profile measured along the *dashed white line* within the *boxed area*. Contrary to heat shock, only the eIF4E1 variants were able to co-localize with both SGs and PBs (**a**, **b**). The eIF4E2 protein variants (**c**–**e**) co-localized only with PBs. eIF4E3_A (**f**) was present only in SGs, and eIF4E3_B (**g**) co-localized with neither SGs nor PBs. Approximately 50 cells transfected with either vector were observed in two independent biological replicates. *Scale bar*, 20 µm

Exposure to oxidative stress and heat shock are known to provoke a multiplicity of cellular responses, including the localization of eIF4E1 to mRNP foci. Even if the cellular response to both of these stresses displays many similarities in general, many differences can be found in detail. We wanted to investigate changes in the distribution of proteins belonging to human eIF4E subfamilies into cytosolic granules upon arsenite treatment, a known oxidative stressor, and to compare that with high-temperature treatment. Cells ectopically producing GFP-tagged eIF4E1 and eIF4E3 proteins rendered identical results upon 40 min of arsenite treatment as well as after their exposure to heat (compare Figs. 3, 5). As previously assessed for heat-induced stress, we observed that eIF4E3_A localized to SGs exclusively (Fig. 5f), whereas the eIF4E3_B isoform was missing in both PBs and SGs (Fig. 5g). Strikingly, upon arsenite treatment and in vivid contrast with heat-shocked cells, GFP-eIF4E2 isoforms were recruited to PBs only, whereas they were completely absent in SGs (Fig. 5c–e). We observed identical results for all eIF4E2 protein isoforms tested. This finding clearly suggests distinct functions of eIF4E2 protein in a cellular response to high temperature or arsenite treatment and indicates a different SG composition in the U2OS cell line upon each of the two stresses.

**Fig. 6:**
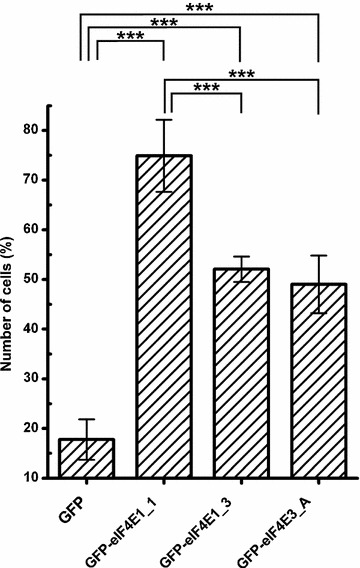
eIF4E1_3 and eIF4E3_A isoforms are less prone to form SGs than the prototypical eIF4E1_1. eIF4E1_1, eIF4E1_3, and eIF4E3_A proteins were ectopically produced in fusion with GFP from the same vector in U2OS cells. Nineteen hours post-transfection, the cells were treated with 1 mM sodium arsenite for 40 min, and those forming SGs were counted and plotted as a fraction of all transfected cells. *Error bars* indicate differences among three independent experiments, in which approximately 100 of the transfected cells were assessed. We applied Chi square test to analyze differences between number of cells forming stress granules among all transfected cells expressing individual eIF4E proteins or a GFP control. GFP-eIF4E1_1, GFP-eIF4E1_3, GFP-eIF4E3_A were compared to control pGFP and to each other by post hoc Chi square test with Bonferroni correction for multiple testing. Exept the GFP-eIF4E1_3 x GFP-eIF4E3_A pair, all other differences were statistically significant (p values <0.0001, marked with *asterisk*)

We noticed that cells transfected with GFP-eIF4E1_3 expression plasmid were less prone to form GFP-eIF4E1_3-positive SGs than cells producing its GFP-eIF4E1_1 counterpart. We quantified the number of cells in which a particular eIF4E protein variant co-localized with SGs upon arsenite treatment, and we plotted this number as a fraction of total number of all transfected cells counted (Fig. 6). This analysis revealed that 75 % of cells ectopically producing the prototypical GFP-eIF4E1_1 formed eIE4E-positive SGs, whereas the number of cells forming SGs dropped to 52 and 49 % for those ectopically producing GFP-eIF4E1_3 and GFP-eIF4E3_A, respectively. Intriguingly, in this regard, the eIF4E1_3 variant behaves similarly to isoform eIF4E3. This is the first evidence indicating possible differences in the activity and/or function between the distinct eIF4E1 variants. As a negative control, we employed cells expressing GFP alone, which co-localized with SGs in 18 % of the cells. A similar effect to the latter has been described elsewhere, with transfection-induced cellular stress being the likely cause [40].

We also investigated the effect of the ectopic expression of the eIF4E1 and eIF4E2 protein isoforms on their localization to PBs. Contrarily to what we observed for SGs, we did not determine any significant difference in their abilities to promote PB formation both in untreated cells and upon arsenite stress. The fraction of cells forming GFP-eIF4E-positive PBs (4E-PB-C) upon transfection with the corresponding expression vector 24 h after transfection was as follows (the first number reflects the % of 4E-PB-C from all transfected cells in normal condition, and the number after the slash indicates the % of 4E-PB-C from all transfected cells under arsenite stress): eIF4E1_1 28.3/54.5; eIF4E1_3 39.5/51.5; eIF4E2_A 37.7/60; eIF4E2_C 35.3/53.2 and eIF4E2_CRA_a 34.5/61.2. Although an increase in the number of cells showing eIF4E containing PBs was evident under arsenite stress, there were no significant differences between any of the eIF4E1 and/or eIF4E2 variants.

### Human endogenous eIF4E1 and eIF4E2 behave under stresses similarly as their GFP-tagged counterparts

**Fig. 7:**
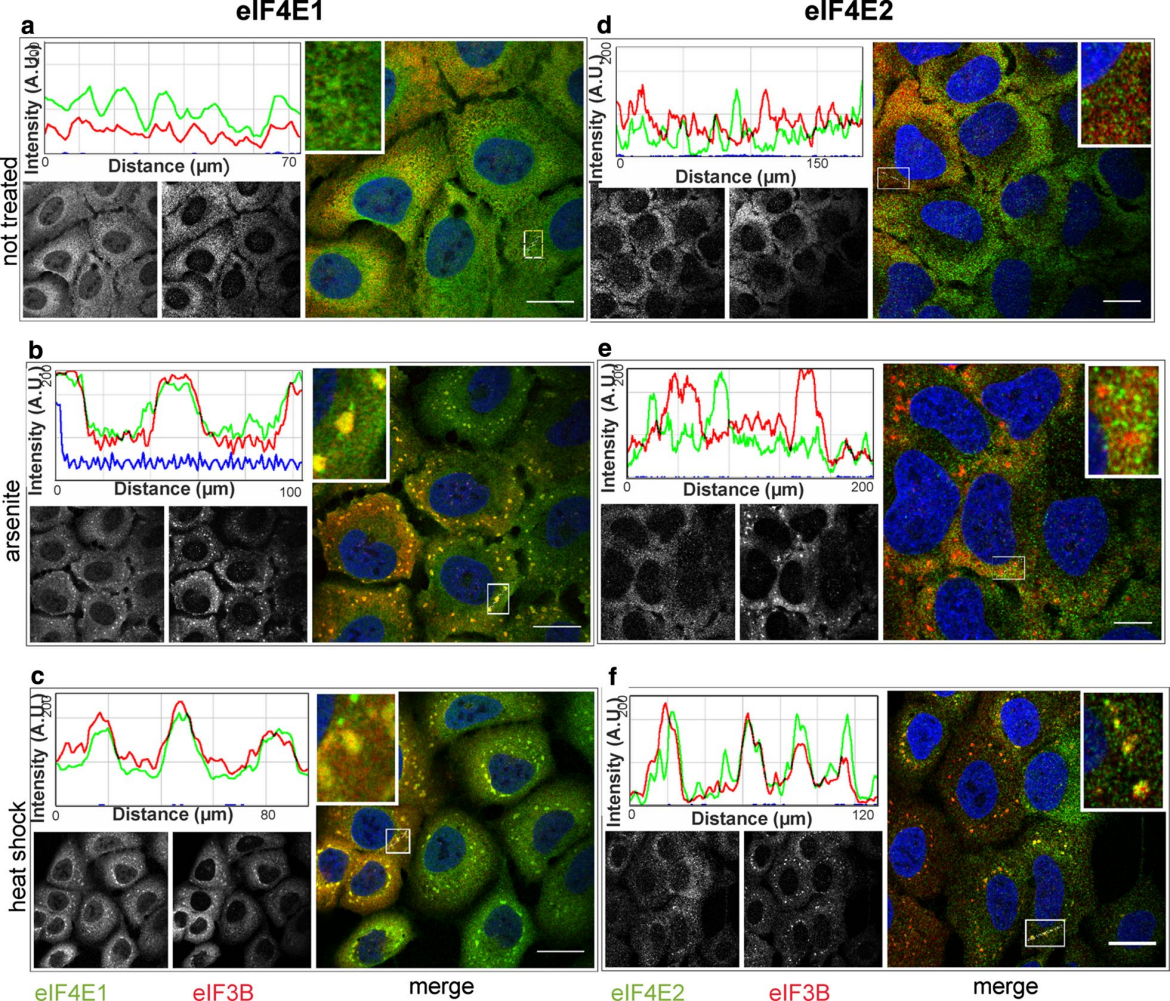
eIF4E2 becomes a component of SGs during heat shock but not in a arsenite stress. U2OS cells were grown in stress-free conditions (**a**, **d**), treated with sodium arsenite (B, E) or exposed to heat (C, F), and then stained with antibodies against eIF4E1 (**a**–**c**, *green*), eIF4E2 (**d**–**f**, *green*) and eIF3B (SG marker, *red*). Co-localization of the particular eIF4E with SGs is demonstrated by merge (on the *right of each panel*) and the intensity profile along the *dashed white line* in the *boxed area* (shown again in 3× magnification on the *right side* of the corresponding intensity profile). In agreement with experiments based on GFP-tagged proteins, the immunostaining of endogenous eIF4E1 and IF4E2 shows a specific recruitment of eIF4E2 to SGs during heat stress (**f**). *Left B/W image* in each panel shows localization of the corresponding endogenous eIF4E protein, *right B/W image* in each* panel* shows eIF3B. Noticeable fractions of both eIF4E1 and eIF4E2 are visibly localized in the cellular nuclei. Nuclei were stained with DAPI (*blue*). Approximately 50 cells were observed in each parallel. *Scale bar*, 20 µm

To further control our experiments based on GFP-tagged proteins, we decided to confirm the localization of the eIF4E factors to SGs by investigating endogenous eIF4E proteins during heat shock and arsenite stress. Difficulties were encountered in sourcing an antibody specific to eIF4E2 and eIF4E3. Regarding eIF4E2, out of the nine different commercially available antibodies tested, only one returned satisfactory results in both immunostaining and western blot analyses. In the case of eIF4E3, our attempts to obtain a reliable antibody were unsuccessful. We confirmed that endogenous eIF4E2 did not co-localize with SGs in arsenite-treated cells (Fig. 7e), whereas it remained co-localized with a substantial fraction of SGs in heat-shocked cells (Fig. 7f). No SGs and thus no co-localization of eIF4E1 and/or eIF4E2 with the SG marker, eIF3B, were detected in stress-free conditions (Fig. 7a, d). This result is in complete agreement with the results obtained using the ectopic expression of the corresponding GFP-tagged eIF4E isoforms and may reflect a predicted role of eIF4E2 as a repressor that binds to a specific subset of mRNAs [21–24]. It likewise indicates possible differences in mRNA content between SGs in arsenite and heat-treated cells.

### eIF4E3_A interacts with eIF4G1, eIF4G3 and PABP1

**Fig. 8:**
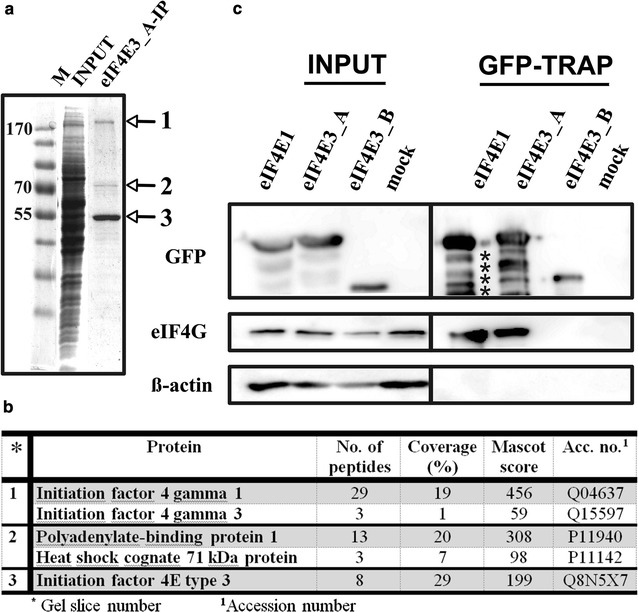
eIF4G interacts with eIF4E3_A but not with eIF4E3_B. **a** Coomassie blue stained gel demonstrating immunoprecipitation of the ectopically expressed GFP-eIF4E3_A from the HEK293 cell lysate using a GFP-Trap approach. *M* PageRuler™ Prestained Ladder (Thermo Scientific); *INPUT* whole cell lysate; eIF4E3_A-IP proteins co-immunoprecipitating with GFP-eIF4E3_A. **b** MS analysis of the proteins co-immunoprecipitating with eIF4E3_A. Gel slices are numbered as in panel A. **c** Western blots of proteins co-immunoprecipitating with eIF4E1_1, eIF4E3_A and eIF4E3_B transiently expressed in GFP fusion in HEK293 cells using GFP-Trap agarose beads (GFP-TRAP). Membranes were developed with anti-GFP (detecting eIF4E-GFP fusion proteins), anti-eIF4G and anti-β-actin antibodies. INPUT lines including β-actin and eIF4G served as a loading control. Lysate from non-transfected HEK293 cells was used as a negative control (mock). The absence of detectable β-actin on GFP-Trap beads shows no contamination of non-specifically bound proteins in the samples

**Fig. 9:**
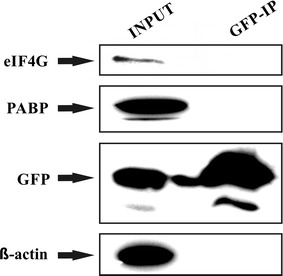
Control immunoprecipitation experiment does not reveal any non-specific interaction between GFP and eIF4G or PABP: HEK293 cells transiently transfected with a control expression vector pEGFP-C1 were lysed 24 h post-transfection and the lysate was subjected to immunoprecipitation using GFP-Trap approach. Western blots were developed with anti-eIF4G, anti-PABP, anti-GFP and anti β-actin antibodies. The results clearly show that while all the proteins tested were present in the lysate, only GFP remained bound to the resin upon GFP-Trap immunoprecipitation

**Fig. 10:**
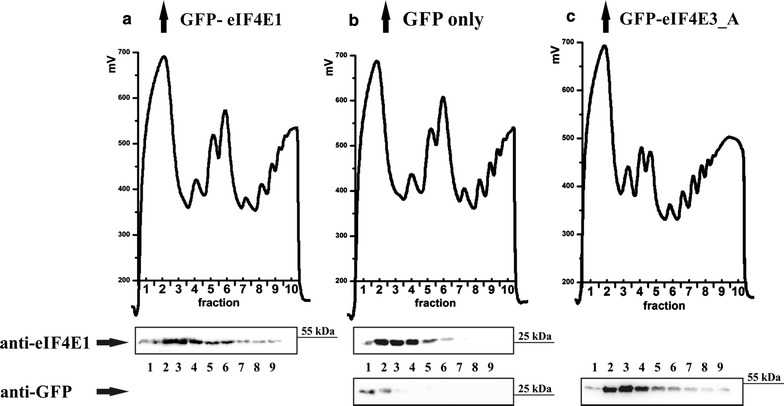
Polysome profile analysis revealed that eIF4E3_A associates with translation intiation complexes and light polysome fractions. GFP-eIF4E1 (**a**) and GFP-eIF4E3_A (**c**) stably expressed in HEK293 cells are distributed along polysome profiles similarly to endogenous eIF4E1 (**b**). To evaluate possible influence of a GFP-fusion tag, a polysome profile analysis from HEK293 cells stably expressing GFP alone was performed (**b**). Western blots of the first nine fractions were probed either with anti-eIF4E1 antibody (**a**, **b**) or anti-GFP antibody (**b**, **c**). Highest amounts of GFP-eIF4E1, GFP-eIF4E3_A and endogenous eIF4E1 were detected from the end of the loading peak to the light polysomes, whereas GFP protein alone was detected in the early loading peak exclusively. Loading peak (≈fractions 1–3); 40S, 60S and 80S (≈fractions 4–6 in **a**, **b** and 3–5 in **c**); light and heavy polysomes (≈fractions 7–10 in **a**, **b** and 6–10 in **c**)

The recruitment of eIF4E3_A to SGs, observed in both heat-shocked and arsenite-treated cells, suggested that eIF4E3_A can act similarly to eIF4E1 with regard to its cellular function. To confirm this assumption, we took advantage of GFP-tagged eIF4E3_A and high selectivity and tight binding of GFP to the GFP-Trap agarose resin, and searched for the eIF4E3 interacting partners by GFP-Trap pull-down, followed by mass spectrometry. The capture of proteins of interest with GFP–Trap_A beads yielded only three prominent bands on polyacrylamide gel stained by Coomassie Blue (Fig. 8a). Bands were excised, and the corresponding protein identities were obtained by MS analysis (Fig. 8b), which revealed the presence of human eIF4E3_A in a complex with eukaryotic translation initiation factor 4G (eIF4G1, isoform 1) and polyadenylate-binding protein 1 (PABP1). Both members of the eukaryotic translation initiation complex were detected with high score and hit coverage. A peptide specific for eIF4G protein isoform 3 (eIF4G3) was also identified, leading us to conclude that both eIF4G1 and eIF4G3 interact with eIF4E3_A in vivo. Significantly, no visible band was observed at the position expected for eIF4G in the eIF4E3_B-IP gel lane (data not shown). Furthermore, the MS analysis of the eIF4E3_B-IP gel area corresponding to 70 kDa-sized proteins retrieved only HSP 70 1A/1B and Heat shock cognate 71 kDa, whereas PABP1 was absent in this case. To confirm that eIF4G binds eIF4E3_A, but not eIF4E3_B, we performed a series of GFP-Trap experiments followed by western blot detection using HEK293 cell lines stably expressing one of the eIF4E1, eIF4E3_A or eIF4E3_B isoforms. As shown in Fig. 8c, only eIF4E1 and eIF4E3_A co-precipitated with eIF4G in a GFP-Trap immunoprecipitation experiment. No detectable amount of β-actin was seen in the GFP-Trap pull-down samples, confirming sufficient washing and high specificity of the GFP-Trap resin. We also did not observe any non-specific binding of eIF4G and/or PABP to GFP itself using a GFP-Trap pull-down technique (Fig. 9). Reverse immunoprecipitation using endogenous eIF4G as bait proved the interaction of eIF4G with GFP_eIF4E1_1 and GFP_eIF4E3_A. As expected, no interaction of endogenous eIF4G and eIF4E3_B was detected (data not shown). We wanted to test whether eIF4E3_A is loaded to the translation initiation complexes and performed analysis of polysome profiles from HEK293 cells stably expressing each of GFP-eIF4E1, GFP-eIF4E3_A and GFP alone. The analysis revealed a similar distribution of GFP-eIF4E1, GFP-eIF4E3_A and endogenous eIF4E1 along the polysome profiles, whereas GFP protein alone was observed in the loading peak fractions only (Fig. 10). These experiments convincingly suggest that human eIF4E3_A but not eIF4E3_B is involved in translation initiation as a part of the eIF4F translation initiation complex.

## Discussion

Several chemical and physical insults are known to have a significant effect on cellular translation. Exposure to heat and oxidative stress inducers provokes a multiplicity of cell responses, including the relocalization and sequestering of initiation factor eIF4E1 to mRNP foci. In the present study, we investigated the effect of heat shock and arsenite treatment on the changes of the subcellular localization of canonical human eIF4E1 and its less investigated isoforms.

Alternative transcription initiation and post-transcriptional maturation events generate multiple distinct transcripts from a single gene. It is estimated that 95–100 % of human multi-exon genes undergo alternative splicing [60, 61], which (i) increases the amount of protein isoforms, leading to changes in enzymatic properties and the spectrum of interacting partners, and (ii) may influence protein localization in the cell, along with other protein features [62]. Our study is thoroughly focused on seven human eIF4E protein isoforms belonging to all three eIF4E protein classes, which further differ in their N- and C-termini (Fig. 1). Six out of seven corresponding transcript variants were cloned from one cDNA derived from B-cell precursor leukemic cell line REH, underscoring the coexistence of distinct splice variants of one gene in human cells. The present study assessed the ability of distinct eIF4E variants to localize into RNA granules, i.e., SGs and PBs, and the ability of eIF4E3 variants to associate with the translation initiation machinery.

Proteins eIF4E1_1 and eIF4E1_3 served for comparison with protein variants from less studied eIF4E2 and eIF4E3 classes. Notably, upon arsenite treatment, cells transfected with the eIF4E1_1 construct formed eIF4E1-positive SGs more readily than those producing eIF4E1_3 proteins (Fig. 6). As a result of alternative splicing, transcript variant eIF4E1_3 possesses a longer N-terminal part than the prototypical isoform 1. It remains, however, challenging to explain how distinct N-termini might influence the localization of both eIF4E1 isoforms to SG. Changes in protein stability and folding, modified affinity to their binding partners and different posttranslational modifications might account for the reasons. This result emphasizes the importance of careful differentiation even highly related protein variants generated from alternatively spliced mRNA transcripts when pursuing functional studies.

In this study, the eIF4E2 protein family was represented by eIF4E2_A, eIF4E2_C and eIF4E2_CRA_a variants. All of them were recruited to PBs and were absent in SGs in cells undergoing arsenite-driven oxidative stress (Fig. 5c–e). This distinct localization pattern clearly distinguishes eIF4E2 from eIF4E1 and eIF4E3, although all these proteins are related at the sequence and structural levels. Moreover, it suggests that eIF4E2, unlike prototypical eIF4E1, is functionally unimportant for SG assembly [34]. This finding enriches the intriguing spectrum of roles attributed to eukaryotic translation factor 4E2, which is also known to act as a translational repressor of specific mRNAs [21–24]. It is conceivable that the exclusive recruitment of eIF4E2 to PBs may reflect its role in processes as translation repression and mRNA storage or decay. Notably, we observed eIF4E2 co-localizing with a number of SGs in heat-shocked U2OS cells containing both ectopically expressed GFP-tagged eIF4E2 and/or expressing entirely its endogenous levels (Figs. 3c–e, 7f). We detected the co-localization of eIF4E2 with a substantial portion—but not all—of eIF3B-specific foci in the same cell. This result provides clear evidence that the protein composition of SGs can vary over different types of stresses. Some other examples of variations in SG protein content, depending on the stress type and/or its severity, have been reported. For instance, selenite elicits the assembly of SGs, which are smaller than arsenite-induced SGs and contain a slightly modified spectrum of proteins, lacking, e.g., eIF3B, which is otherwise an established SG marker [39, 41].

The eIF4E2_A protein isoform contains five C-terminal leucines, the spacing of which might fulfill the criteria of a nuclear export sequence (NES). The corresponding sequence is, however, missing in the eIF4E2 protein isoforms C and CRA_a because these utilize different C-terminal exon composition (Fig. 1). Using naturally occurring protein variants, we showed that eIF4E2_C and eIF4E2_CRA_a did not demonstrate increased accumulation in cell nuclei in comparison to prototypical isoform eIF4E2_A (Figs. 3, 4, 5), thus confirming that the C-terminal extension of eIF4E2_A does not contain a NES [63].

We did not observe any significant differences among all eIF4E1 or eIF4E2 protein isoforms tested with respect to their ability to be recruited to PBs both in normal conditions and under arsenite stress.

One of the less studied eIF4E family members, eIF4E3_A, displays both cytoplasmic and nuclear localization {(Figs. 3, 4, 5); [20]}. Here, we show that in human cells, the eIF4E3_A isoform exhibits a unique stress response, being recruited to SGs in both heat-shocked and arsenite-treated cells but never localizing to PBs (Figs. 3f, 5f). The localization of human eIF4E3_A to SGs led us to hypothesize that this isoform may complement the roles of eIF4E1 in the process of translation initiation. This assumption is additionally advocated by a study reporting on neural transmembrane receptor DCC in a complex with many components of the active translational apparatus, including eIF4E3 [64].

To test this hypothesis, we performed an immunoprecipitation of GFP-tagged eIF4E3_A, followed by an MS analysis of bound proteins (Fig. 8). We detected two eIF4G isoforms along with poly(A)-binding protein 1 (PABP1) with a high score and hit coverage strongly suggesting that at least a fraction of eIF4G trapped in our experiment arose from translating ribosomes. This is the first report indicating the role of human eIF4E3 in translation initiation. It is also a contribution to the very recent and somewhat contradictory results reported by two research groups using the same constructs to express mouse eIF4E3 in mouse and/or human cells. Although both human endogenous and ectopically expressed mouse eIF4E3 proteins were able to bind the ^7^mG cap efficiently during cap-column chromatography, the interaction of ectopically expressed mouse eIF4E3 with eIF4G was not detected in mouse NIH 3T3 fibroblasts [20] but can apparently be observed in the HLY-1 diffuse large B-cell lymphoma cell line [29]. In the latter experiment, both mouse eIF4E3 and its C-terminally truncated version eIF4E3-D199 co-precipitated two members of the eIF4F complex (eIF4G and eIF4A) efficiently, even if the ability of mouse eIF4E3-D199 to bind the ^7^mG cap could not be detected [20, 29]. In the original paper by Joshi et al. who pioneered the classification of eIF4E protein isoforms, mouse eIF4E3 was also shown to interact with eIF4G peptide in a pull-down assay, attesting to its functional importance [19]. The observed in vivo interaction of eIF4E3 with eIF4G is in agreement with eIF4E3 primary structure because it contains most of the conserved amino acid residues known to be important for the eIF4E1-eIF4G interaction, including P38, V69, L135 and the invariant W73. By contrast, eIF4E3 binds eIF4G peptide 40 times less efficiently than eIF4E1 in vitro; conserved H37, Q40 and L131 residues known to be involved in eIF4E1-eIF4G interaction are missing, and a2′ helix maps away from the eIF4G peptide binding site [7, 20]. Taking all that together, it is to be considered what portion of the eIF4E3-eIF4G complex genuinely takes part in the translation initiation, in which cellular environment such an interaction may occur and what role differences between mouse and human eIF4E3 and eIF4G proteins may play.

Mouse and human eIF4E3 proteins are not identical. Mouse eIF4E3—similarly to its rat homologue—is only 207 amino acids long, whereas its human counterpart spans 224 amino acid residues. It appears that rodent eIF4E3 proteins are more derived and lack the N-terminal extension, which is present in other known eIF4E3 sequences. Human eIF4E3 further differs from the mouse homologue in six amino acid residues with substitutions of uncharged or neutral amino acid residues to polar or charged ones, respectively (Fig. 1). An explanation of the possible differences in the binding properties of both proteins remains, however, an open matter.

In mice, the expression of eIF4E3 is fairly limited; in fact, eIF4E3 mRNA was reported only in skeletal and heart muscles, lungs and spleen [19]. In humans, the eIF4E3_A protein was detected in several hematopoietic cell types [20]. A systematic human proteome study summarized in The Human Protein Atlas detected eIF4E3 at high or medium expression levels in 11 out of 79 analyzed normal tissue cell types [65]. Moreover, eIF4E3 is sparsely covered by deposited full-length cDNA or EST sequences. There are 156 ESTs available in the UniGene Database, supporting the existence of eIF4E3 in human cells; however, only one of them evidences the eIF4E3_A protein isoform (DR159502.1). The clone originates from human embryonic stem cells differentiated to an early endodermal cell type; this could explain our finding of eIF4E3_A in HEK293 cells, which are derived from an embryonic kidney.

The truncated isoform of human eIF4E3, eIF4E3_B, lacks a significant part of the eIF4G-binding consensus, including the W73 residue. In this study, we demonstrated that eIF4E3_B neither localized to SGs or PBs nor bound the scaffold protein eIF4G (Figs. 3g, 5d, 8c). This outcome is in agreement with the reported importance of the conserved motif containing a W73 residue, which is critical for eIF4E1 interaction with both eIF4G and 4E-BPs and, consequently, for eIF4E1 localization to both SGs and PBs [7, 8, 17]. To elucidate the possible function of eIF4E3 in humans, determination of protein abundance and tissue/developmental specificity for each eIF4E3 protein isoform is needed.

**Fig. 11:**
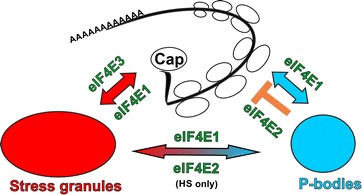
Possible roles of eIF4E1, eIF4E2 and eIF4E3 in translation inititation and mRNA repression. Abundant and tightly regulated eIF4E1 plays an important role both in translation initiation and translation repression and therefore localizes to sites of active translation, PBs and SGs. The major role of eIF4E2 is in translation repression and therefore localizes mainly to PBs. Different composition of SGs as a consequence of different stresses and dynamic flux of molecules between PBs and SGs is suggested by the presence of eIF4E2 in SGs after heat shock but not sodium arsenite treatment. Low abundant eIF4E3_A may serve as a keeper of basal translation initiation which is not regulated by 4E-BP pathway and is not involved in mRNA repression and decay pathways. eIF4E3_A thus localizes to SGs but never to PBs upon stresses. Colour coding is the same as in other figures: eIF4Es are in green, PBs are in* blue* and SGs are in* red*. For simplification, we do not include other eIF4E1 regulatory pathways and shuttling of all three eIF4Es between cytoplasm and nucleus

Results presented in this study, current knowledge about links between translation and PBs or SGs formation and known data about the eukaryotic translation initiation factors belonging to the eIF4E family allow us to suggest a speculative model of distinct function of different eIF4Es in human cells (Fig. 11). PBs contain non-polyadenylated mRNAs, components of mRNA repression pathways, mRNA decay machinery and numerous RNA-binding proteins and therefore are suggested to be sites of mRNA repression and degradation [41, 43]. SGs, on the other hand, contain polyadenylated mRNA, small ribosomal subunits, most translation initiation factors, cytoplasmic poly(A)-binding protein and a varying set of RNA-binding proteins and thus are considered as sites of accumulated stalled translation initiation complexes, which are formed as a response to various stress conditions [41]. Several studies evidenced that mRNAs within SGs are in a continuous flux and remain in a highly dynamic equilibrium with polysomal mRNA [41, 46, 66]. In our model, a tightly regulated eIF4E1 [67, 68] shuttles between sites of active translation, PBs and as a response to stress insult also SGs [41]. eIF4E2 is generally considered as a protein mainly involved in translation repression [21–24], which however can associate with translating ribosomes in human cells during hypoxia [25, 26]. This is in agreement with our observation that all three eIF4E2 variants tested (eIF4E2_A, eIF4E2_C and eIF4E2_CRA_a) localize to PBs both in normal conditions and in arsenite-induced stress. Intriguingly, eIF4E2 appears in some, but not all, stress granules as a result of heat shock. One of the possible explanations might be, that translation of small subset of mRNAs could be facilitated by eIF4E2 even in normal conditions in human cells, similarly as in nematode [27], and their translation initiation might be sensitive to elevated temperature but not to arsenite treatment. We favour another possible explanation, that human SGs composition is different in heat shock and arsenite treated cells [41] and thus the dynamic flux and protein and mRNP exchange between PBs and SGs allow trapping of eIF4E2 (or mRNPs containing eIF4E2) in heat shock-specific SGs, but not arsenite-specific SGs, due to possible protein–protein interactions. The mRNP exchange between PBs and SGs is facilitated by docking PBs with SGs, which has been reported in cells treated with sodium arsenite or the mitochondrial poison FCCP but never in cells subjected to heat shock [41, 46, 59]. In contrast, we observed association of SGs and PBs in U2OS cells after heat shock frequently, regardless if they overexpressed any of the eIF4E proteins tested or not (Fig. 3). The reason might be that we optimized heat shock conditions to maximize SG formation and well controlled each experiment by measuring temperature with a microprobe directly attached to coverslip with growing cells. eIF4E3_A does not bind 4E-BP proteins [19] and thus is probably less tightly regulated than eIF4E1. eIF4E3_A is also much less abundant in cells than eIF4E1 and eIF4E2 as inferred from northern blot analyses [19], low occurrence of ESTs corresponding to eIF4E3_A in databases (this text) and apparent difficulties to detect endogenous eIF4E3_A by western blot [19, 29 and our unpublished results]. In our experiments, eIF4E3_A did not localize to PBs but readily moved to SGs upon arsenite stress and heat shock. eIF4E1 is an abundant and dominant cap-binding translation initiation factor, which is responsible for most of the cellular translation initiation. eIF4E1 is tightly regulated and acts also as an important regulator on itself [67, 68]. We can speculate that eIF4E3_A carries out basal translation initiation when eIF4E1 is repressed and/or eIF4E3_A secures translation of specific subset of mRNAs which should not respond to changes directed by cellular pathways controlling eIF4E1 function. This would explain the low abundance of eIF4E3_A in most tissues [19] because, as we show here, human eIF4E3_A can readily associate with translation initiation complexes, but its inability to bind 4E-BPs might allow eIF4E3 to escape from the overall cellular translation control and thus ruin the whole eIF4E1 regulatory network. Besides eIF4E1 and eIF4E3, eIF4E2, which is quite abundant in all tissues [19], presumably mainly functions in repressing specific cellular mRNAs, which corresponds with its localization to PBs both in normal and stressed cells.

## Conclusion

To our knowledge, this is the first investigation evaluating the cellular redistribution of all human members of the eIF4E protein family upon arsenite or heat stresses. We showed that eIF4E3_A localizes to SGs but not PBs upon heat shock or arsenite stress. This finding allowed us to speculate about the function of human eIF4E3_A in translation initiation. We obtained some evidence about that via the demonstration of the in vivo interaction of eIF4E3_A with eIF4G1, eIF4G3 and PABP1 and the eIF4E3_A loading to monosome and light polysome fractions. Contrary to this finding, the truncated eIF4E3 isoform, eIF4E3_B showed no localization to SGs and no binding to eIF4G. We extended our study on relocalization of eIF4E isoforms to cytoplasmic mRNP granules to some of their variants resulting from the alternative pre-mRNA splicing. Surprisingly, we found some differences in the ability of eIF4E1_1 and eIF4E1_3 to form stress granules in response to cellular stresses. This initial finding might be of general importance because it provides one of few known pieces of evidence of the assumed functional differences between human protein variants arising from alternatively spliced transcripts. We also showed that eIF4E2 may exhibit distinct functions under different stresses as it readily relocalizes to P-bodies during arsenite and heat stresses, whereas it is redirected to stress granules only upon heat shock, which also indicates the variable protein content of SGs as a consequence of different stress insults. This is supported by our observation that PBs associate with SGs in heat stressed cells. The comparison of the cellular distribution of three naturally occurring variants of eIF4E2 allowed us to confirm that the eIF4E2_A protein isoform does not contain a nuclear export sequence (NES), as could be hypothesized from its C-terminal leucine-rich motif. Last but not least, we developed a reproducible method for inducing heat shock in mammalian cell cultures.

## Additional file

10.1186/s12867-016-0072-x Precise heat shock optimization and accurate calibration of temperature measurement for efficient and reproducible SGs formation (PDF 132 kb).

## Abbreviations

eIF4E: eukaryotic translation initiation factor 4E
eIF4E1, eIF4E2 and eIF4E3: isoforms of the eukaryotic translation initiation factor 4E
eIF4G: eukaryotic translation initiation factor 4G
eIF4F: a complex composed of eIF4E, eIF4G and eIF4A
PABP: polyadenylate-binding protein
PB: processing body
SG: stress granule
