# Supplementary material for: Distinct recruitment of human eIF4E isoforms to processing bodies and stress granules
Source: BMC Mol Biol. 2016 Aug 30;17(1):21. doi: 10.1186/s12867-016-0072-x (PMC5006505; doi:10.1186/s12867-016-0072-x)
Supplement: Supplementary file 1 — 10.1186/s12867-016-0072-x Precise heat shock optimization and accurate calibration of temperature measurement for efficient and reproducible SGs formation (PDF 132 kb). [file 12867_2016_72_MOESM1_ESM.pdf]

Additional figure 1. Precise optimization and accurate calibration of temperature measurement were essential for efficient and reproducible SGs formation.

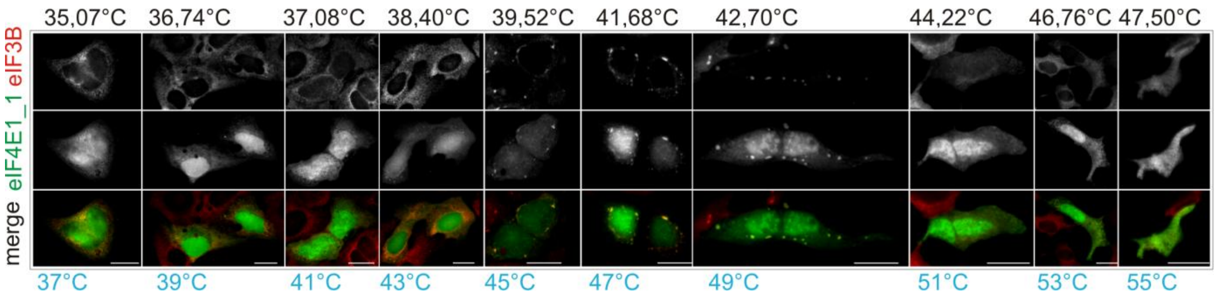

U2OS cells were grown on a glass coverslip and transfected with an expression vector coding for the GFP-eIF4E1_1 fusion protein. Nineteen hours post-transfection, the cultivation dish was placed on a pre-heated thermoblock, and a submersible temperature probe was placed into the medium in direct contact with the coverslip. The medium was subsequently exchanged with another one pre-warmed to the required temperature. The dish containing the coverslip was closed, covered with a plastic box and incubated on the thermoblock for 30 min. Temperatures of the pre-warmed medium and the thermoblock were set experimentally to reach the required temperature on the coverslip. The temperature was read at the beginning and at the end of the treatment. The calculated mean temperature values are displayed above each panel. The corresponding temperature values that were set on the thermoblock are shown below each panel in blue. Following heat shock, cells were fixed and assessed for eIF3B-stained SGs. The temperature gradient clearly shows that only a narrow range of high-fevered temperatures was suitable for the efficient induction of SG formation. Scale bar, 20 µm.
